# Pim3 up-regulation by YY1 contributes to diabetes-induced cardiac hypertrophy and heart failure

**DOI:** 10.22038/ijbms.2024.78688.17016

**Published:** 2025

**Authors:** Xiao-Ping Jin, Yi-Fei Ren, Li-Guo Wang, Hao Xie, Lu Huang, Juan Zhang, Zuo-Ying Hu

**Affiliations:** 1 Department of Cardiology, Nanjing First Hospital, Nanjing Medical University, Nanjing, China; # These authors contributed equally to this work

**Keywords:** Cardiac hypertrophy Diabetes mellitus, Heart failure, Proviral insertion of- Moloney virus 3 kinase, Yin Yang 1

## Abstract

**Objective(s):**

The close relationship of proto-oncogenes to myocardial hypertrophy has long been recognized, and cardiac hypertrophy leads to heart failure (HF). However, whether proviral insertion of Moloney virus 3 kinase (Pim3), a proto-oncogene, contributes to cardiac hypertrophy in diabetes mellitus (DM) remains unknown. This study aims to investigate whether Pim3 is involved in DM-induced cardiac hypertrophy and HF and to elucidate its underlying mechanisms.

**Materials and Methods:**

DM was induced in mice by intraperitoneal injection of streptozotocin. Cardiac function was evaluated by echocardiography, and cardiac hypertrophy was determined through histological analysis, quantitative real-time polymerase chain reaction, and western blotting. Silencing RNA transfection and lentivirus-mediated gene knockdown were performed both in vitro and in vivo. Transcriptional activity was analyzed using chromatin immunoprecipitation and luciferase reporter assay.

**Results:**

Compared with C57BL/6J mice, cardiac hypertrophy and dysfunction were observed in mice with DM. Pim3 mRNA and protein expression were significantly up-regulated in diabetic hearts and high glucose-cultured H9C2 cells. Yin Yang 1 (YY1), which translocated from the cytoplasm into the nucleus under hyperglycemia, bound to the Pim3 promoter and enhanced Pim3 transcriptional activity. Pim3 or YY1 knockdown profoundly reduced cell size and inhibited the mRNA and protein expression of cardiac hypertrophy markers, as well as markedly attenuating myocardial hypertrophy and cardiac dysfunction.

**Conclusion:**

Hyperglycemia induced nuclear translocation of YY1, leading to Pim3 up-regulation, eventually developing into cardiac hypertrophy and HF. Targeting YY1-Pim3 signaling may be a promising therapeutic avenue for DM-induced cardiac hypertrophy and HF.

## Introduction

Cardiac hypertrophy is largely characterized by cardiomyocyte enlargement, increased protein synthesis, fetal gene reactivation, cytoskeleton remodeling, and enhanced fibrosis (1, 2). Cardiac hypertrophy plays a crucial role in the pathogenesis and development of heart failure (HF)(3). Diabetes mellitus (DM) is associated with a 2-4-fold increase in HF risk. Moreover, patients with HF and DM have a worse prognosis than those without DM (4). Although the specific mechanism by which DM causes HF is not well understood, DM-induced cardiac inflammation, hypertrophy, and other factors may contribute (5). Therefore, inhibition of hyperglycemia-induced cardiac hypertrophy might markedly improve cardiac function under DM conditions, and this concept has been recognized in many publications (6-8). 

Proto-oncogenes encode various proteins, including growth factors, protein kinases, and transcription factors. C-Myc and c-Fos are among the best-characterized proto-oncogenes known to modulate myocardial hypertrophy (9). Interestingly, proto-oncogenes are significantly up-regulated under DM conditions and are associated with many DM-induced diseases, such as diabetic nephropathy (10) and diabetic retinopathy (11). We thus hypothesized that proto-oncogenes may also participate in DM-induced cardiac hypertrophy and subsequent HF.

Pim (provirus integration site for Moloney murine leukemia virus) kinases, including Pim1, Pim2, and Pim3, are proto-oncogenes with serine/threonine kinase activity (12). Accumulating evidence suggests that Pim1 exerts protective effects on DM-induced myocardial injury and is down-regulated in diabetic hearts (13-15). Pim2 has also been reported to protect the myocardium under ischemia and high-fat diet conditions (16, 17). Compared with the other two isoforms in this family (Pim1 and Pim2), Pim3 is less studied. In the present study, we found that Pim3 RNA and protein expression were significantly increased, both in diabetic hearts and in high glucose (HG)-treated cardiomyocytes. Pim3 knockdown markedly reduced the hyperglycemia-induced mRNA and protein expression of cardiac hypertrophy markers (atrial natriuretic peptide [ANP] and β-myosin heavy chain [MHC]), attenuated cardiac hypertrophy, and improved cardiac dysfunction. Based on these findings, we propose that Pim3 might be involved in DM-associated cardiac hypertrophy and subsequent HF. Therefore, we aim to gain insights into the underlying mechanisms of Pim3 up-regulation under hyperglycemic conditions.

Yin Yang 1 (YY1), a transcriptional repressor or activator, is ubiquitously expressed and evolutionarily conserved. It is a zinc finger protein with a pivotal role in normal biological processes, such as cell development, differentiation, replication, and proliferation, and several studies have demonstrated its association with pathological conditions (18, 19). A previous study showed that YY1 was significantly up-regulated in cardiac hypertrophy and HF (20). However, the effects of YY1 on Pim3 remain elusive. This study demonstrates that YY1 binds to the Pim3 promoter and enhances Pim3 transcriptional activity under hyperglycemic conditions. We speculate that YY1-Pim3 signaling may play a crucial role in DM-induced cardiac hypertrophy and HF.

## Materials and Methods

***Reagents***

D-glucose (#G7528), D-mannitol (#M4125), and antibodies against β-MHC (#ZRB1308) and ANP (#SAB5700150) were purchased from Sigma-Aldrich (St. Louis, MO, US). Antibodies against Pim3 (#4165), YY1 (#46395), histone H3 (#4499), and β-actin (#3700) were obtained from Cell Signaling Technology (Beverly, MA, US). Wheat germ agglutinin (WGA; #W11261) was purchased from Invitrogen (Carlsbad, CA, US). 

***Cell culture***

H9C2 cells, a clonal line derived from the rat heart, were obtained from the Shanghai Institute of Biochemistry and Cell Biology (Shanghai, China). The cells were cultured in Dulbecco’s Modified Eagle Medium (DMEM) (Gibco-BRL, Rockville, MD, US) containing 5.5 mmol/L D-glucose supplemented with 10% fetal bovine serum (Gibco-BRL), 100 units/ml penicillin, and 0.1 mg/ml streptomycin, in a humidified incubator at 37 ^°^C equilibrated with 5% carbon dioxide and 95% air. To establish HG conditions, H9C2 cells were first incubated in normal glucose (5.5 mmol/l) with minimum essential medium for 24 hr to 70% confluence and then exposed to DMEM containing 33 mmol/L glucose for the indicated time.

***Small interfering RNA (siRNA) transfection***

SiRNAs targeting rat Pim3 (sense: 5′-GCUCUACGACAUGGUGUGUTT-3′; antisense: 5′-ACACACCAUGUCGUAGAGCTT-3′), YY1 (sense: 5′- CCAAGAACAAUAGCUUGCCCUCAUA-3′; antisense: 5′- UAUGAGGGCAAGCUAUUGUUCUUGG-3′), and scrambled siRNA (sense: 5′- CCAAACAAUAGCUUGCCCUCAGAUA-3′; antisense: 5′- UAUCUGAGGGCAAGCUAUUGUUUGG-3′) were synthesized by RiboBio Co. Ltd. (Guangzhou, China). H9C2 cells were transfected with 100 nM siRNA using Lipofectamine RNAiMAX reagent (Invitrogen) in accordance with the manufacturer’s protocol. 

***Animals***

Eight-week-old wild-type (WT) male C57BL/6J mice (20-22 g) were obtained from GemPharmatech LLC. (Nanjing, Jiangsu, China) and housed in a 12-hr/12-hr light/dark cycle at 23 ^°^C±2 ^°^C with *ad libitum* access to food and water. Type 1 DM was induced by daily intraperitoneal injection of 50 mg/kg streptozotocin (Sigma-Aldrich) dissolved in 0.05 mol/l citrate buffer (pH 4.5) for five consecutive days after 5-6 hr of fasting. Mice with a blood glucose concentration >300 mg/dl at three weeks after the final streptozotocin injection were diagnosed with DM (21). The Institutional Animal Care and Use Committee of Nanjing Medical University (Nanjing, China) approved the animal protocols. 

***Quantitative realtime polymerase chain reaction (RTqPCR)***

Total RNA was extracted from H9C2 cells and the left ventricular tissue of mice using a Trizol kit (Invitrogen) according to the manufacturer’s protocol. The RNA was then reverse transcribed into complementary DNA (cDNA) using a reverse transcription kit (Takara, Shiga, Japan). The cDNA was amplified using a SYBR Green PCR kit (Takara). The sequences of all primers are listed in Table 1. The expression of the indicated genes was normalized to the reference gene 18S and calculated using the standard 2^-ΔΔCt^ method.

***Evaluation of cardiac function by echocardiography***

The mice were imaged using a Vevo 3100 high-resolution small animal ultrasound system (Fujifilm VisualSonics Inc., Toronto, Ontario, Canada). The testing indices for echocardiography have been described previously (22).

***Preparation of cytoplasmic and nuclear extracts***

For the cytosolic (C)/nuclear (N) fractionation assay, cytoplasmic and nuclear proteins were extracted from H9C2 cells with a Nuclear and Cytoplasmic Protein Extraction Kit (P0027, Beyotime, Haimen, China) according to the manufacturer’s instructions.

***Western blotting***

Protein homogenates were prepared from H9C2 cells and left ventricular tissues and subjected to western blotting in accordance with previously described methods (22). 

***Chromatin immunoprecipitation (ChIP) assay***

The ChIP assay was performed as previously described (23). Briefly, HL-1 cells (immortalized murine cardiomyocytes) (#ZQ0920, Zhong Qiao Xin Zhou Biotechnology Co. Ltd., Shanghai, China) were treated with or without HG (33 mmol/L) and cross-linked with 1% formaldehyde for 10 min at 37 ^°^C. Cross-linking was stopped by the addition of 0.125 M glycine. The cells were washed three times with ice-cold phosphate-buffered saline and kept on ice for 10 min in 25 mM HEPES (pH 7.8), 1.5 mM magnesium chloride, 10 mM potassium chloride, 0.1% Nonidet P 40 (NP-40), 1 mM dithiothreitol, 0.5 mM phenylmethylsulfonyl fluoride, and a protease inhibitor mixture (Roche, Basel, Switzerland). The nuclei were subsequently collected and sonicated on ice to shear the DNA into fragments with an average length of 200 base pairs. After sonication, a chromatin solution (500 µg) was incubated with ChIP-grade antibodies against YY1 (Cell Signaling Technology, Beverly, MA, US) and rabbit immunoglobulin G (IgG; Abcam) overnight at 4 ^°^C. The resulting antibody-bound complexes were precipitated, and the DNA fragments extracted from these complexes were purified using a QIAquick PCR Purification Kit (Qiagen, Dusseldorf, Germany). Pre-immunoprecipitated input DNA was used as a control in each reaction. The purified ChIP DNA samples were analyzed by conventional PCR using forward and reverse primers specific for the mouse Pim3 promoter (forward: 5ʹ-TGAGGGTCTAGGCCACAAAG-3ʹ, reverse: 5ʹ-AACTGTCGTTCCTGAACTGC-3ʹ). IgG was used as the negative control.

***Luciferase reporter assay***

To analyze the regulation between YY1 and Pim3, the wild-type and mutated promoter sequences of Pim3 were respectively cloned into PGL4.10-basic vector by Obio Technology (Shanghai, China). The luciferase reporter assay in HL-1 cells was performed as described previously (24). pCDNA3.1-YY1(mouse)-3×HA (#P16048) and pCDNA3.0 (#P0155) were obtained from MiaoLing Plasmid Platform (Wuhan, Hubei, China). The pRL Renilla luciferase vector (pRL-TK, #E2241) was obtained from Promega (Madison, WI, US). HL-1 cells were plated at a density of 2×10^5^ cells/well in a 24-well plate for 24 hr and transfected using Lipofectamine 3000 (Invitrogen) leveraging luciferase reporter plasmid (Pim3-wt-Luc or Pim3-mu-Luc) and pRL-TK along with YY1 or pCDNA3.0 according to a previously reported protocol (25). The luciferase reporter assay was performed 48 hr later using a Dual-Luciferase Reporter Assay System (#E1910, Promega) according to the manufacturer’s protocol. Specific promoter activity was expressed as the ratio of firefly luciferase activity to Renilla luciferase activity.

***Immunofluorescence assay***

H9C2 cells were analyzed by immunofluorescence as previously described (22). Immunostained H9C2 cells were visualized by confocal laser scanning microscopy coupled with an image analysis system (original magnification ×400; Leica, Wetzlar, Germany).

***Lentivirus injection***

To determine whether Pim3 is involved in the pathogenesis of cardiac hypertrophy in streptozotocin-induced diabetic mice, the *Pim3* and *Yy1* genes were knocked down using short hairpin RNA (shRNA) delivered by lentivirus (LV). LV-Pim3 shRNA (titer: 5.11×10^9^ TU/ml), LV-YY1 shRNA (titer: 4.53×10^9^ TU/ml), and LV-scrambled shRNA (titer: 7.22×10^9^ TU/ml) were generated by Genechem (Shanghai, China). The sequences for Pim3, YY1, and scrambled shRNAs were 5ʹ-GGCATGTCTGCACAAGCAATG-3ʹ, 5ʹ-CGACGGTTGTAATAAGAAGTT-3ʹ, and 5ʹ-CCTAAGGTTAAGTCGCCCTCG-3ʹ, respectively. Eight-week-old male mice (*n*=60) were randomly divided into six groups: WT, DM, DM+physiological saline, DM+LV-scrambled shRNA, DM+LV-Pim3 shRNA, and DM+LV-YY1 shRNA. To verify the efficiency of Pim3 or YY1 lentivirus-mediated knockdown, age-matched male C57BL/6J mice (*n*=24) were allocated to four groups: WT, WT+LV-scrambled shRNA, WT+LV-Pim3 shRNA, and WT+LV-YY1 shRNA. The tail vein was slowly injected with 50 μl LV-packaged Pim3, YY1 or scrambled shRNA with a 0.5-ml insulin syringe. After another 12 weeks, cardiac function and other experiments were performed.

***Enzyme-linked immunosorbent assay (ELISA)***

The blood samples of mice were collected and centrifuged at 2000 rpm for 15 min. The serum was collected and frozen at -80 ^°^C in multiple aliquots until analysis. The serum N-terminal pro-brain natriuretic peptide (NT-proBNP) concentration was measured using a mouse NT-proBNP ELISA kit (#E-EL-M0834, Elabscience, Wuhan, China) following the manufacturer’s protocol.

***Histology***

In all experimental groups, the hearts were excised, fixed with 4% paraformaldehyde solution, and embedded in paraffin. The left ventricle was cut into 5-µm-thick sections along the anterior longitudinal midline as previously described (22). To evaluate the cardiomyocyte size, the sections were stained for WGA (#W11261, Invitrogen). Stained sections were viewed using a fluorescence microscope (Leica; original magnification ×400). The cardiomyocyte area was measured using Image J software (National Institutes of Health, US).

***Statistical analysis***

Data are presented as the mean±standard deviation. Data were compared between the groups using Student’s t-test or one-way analysis of variance (ANOVA) using GraphPad Prism software 8.0 (GraphPad Inc., San Diego, CA, US). When statistical significance was found by ANOVA, Tukey’s *post hoc* test for multiple comparisons was performed. *P*<0.05 was considered statistically significant.

**Table 1:** Primer sequences for β-MHC, ANP, Pim3 and 18S in rat and mouse species

Gene	Species	Primer sequence
β-MHC	Rattus	Forward: CCTCGCAATATCAAGGGAAA
		Reverse: TACAGGTGCATCAGCTCCAG
ANP	Rattus	Forward: GTACAGTGCGGTGTCCAACA
		Reverse: ATCCTGTCAATCCTACCCCC
Pim3	Rattus	Forward: CACTGACTTTGATGGCACCC
		Reverse: ATGCCCAGACGAAGACCAG
18S	Rattus	Forward: GTAAACCCGTTGAACCCCATT
		Reverse: CCATCCAATCGGTAGTAGCG
β-MHC	Mouse	Forward: CTGAAGGGCATGAGGAAGAGT
		Reverse: AGGCCTTCACCTTCAGCTGC
ANP	Mouse	Forward: TACAGTGCGGTGTCCAACACAG
		Reverse: TGCTTCCTCAGTCTGCTCACTC
Pim3	Mouse	Forward: ATGCTGCTCTCCAAGTTCGGCTCCCTGGCG
		Reverse: TCCTGTGCCGGCTCGGGTCGCTCCAGCACC
18S	Mouse	Forward: GGAAGGGCACCACCAGGAGT
		Reverse: TGCAGCCCCGGACATCTAAG

**Figure 1:**
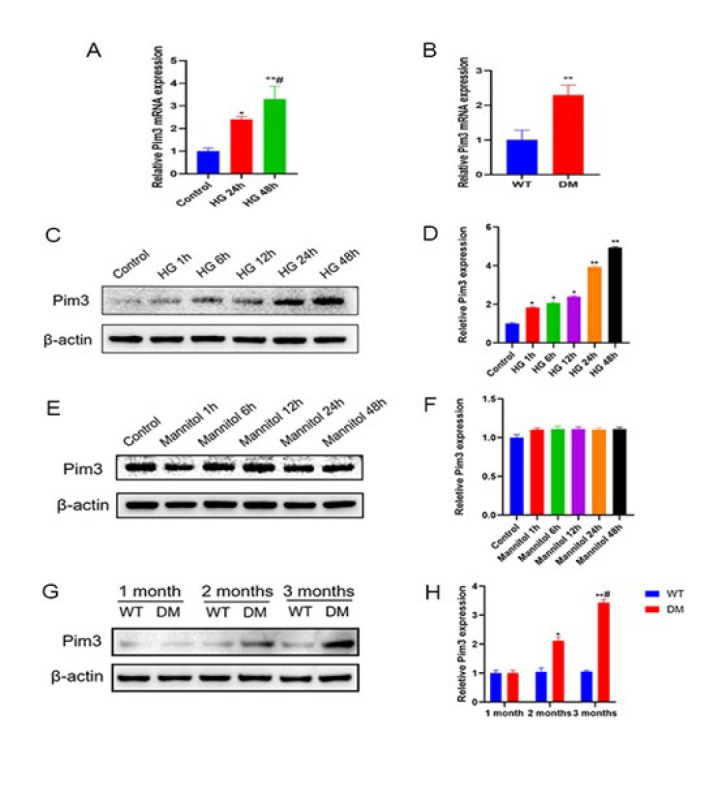
Changes in Pim3 protein expression in cardiomyocytes under hyperglycemic stimulation

(A) H9C2 cells were exposed to HG for the indicated time periods. Pim3 mRNA expression was measured by RT-qPCR. (B) Pim3 mRNA expression was determined by RT-qPCR of heart tissues. 18S expression was used for normalization, and quantitative analysis is shown in the histogram. (C, E) H9C2 cells were exposed to HG medium or mannitol for the indicated times, and the immunoblot bands obtained by western blotting are shown. (G) Hearts were isolated at 1 month, 2 months, and 3 months from WT and diabetic mice. Pim3 protein expression in the heart was measured by western blotting, and the immunoblot bands are shown. (D, F, H) Quantitative presentation of the immunoblots. All data were obtained from at least three independent experiments. *P<0.05, **P<0.01 vs control or WT. ^#^*P<*0.05 vs 24 hr or DM at 2 months

HG, high glucose; Wild-type mice, WT; DM, diabetes mice

**Figure 2:**
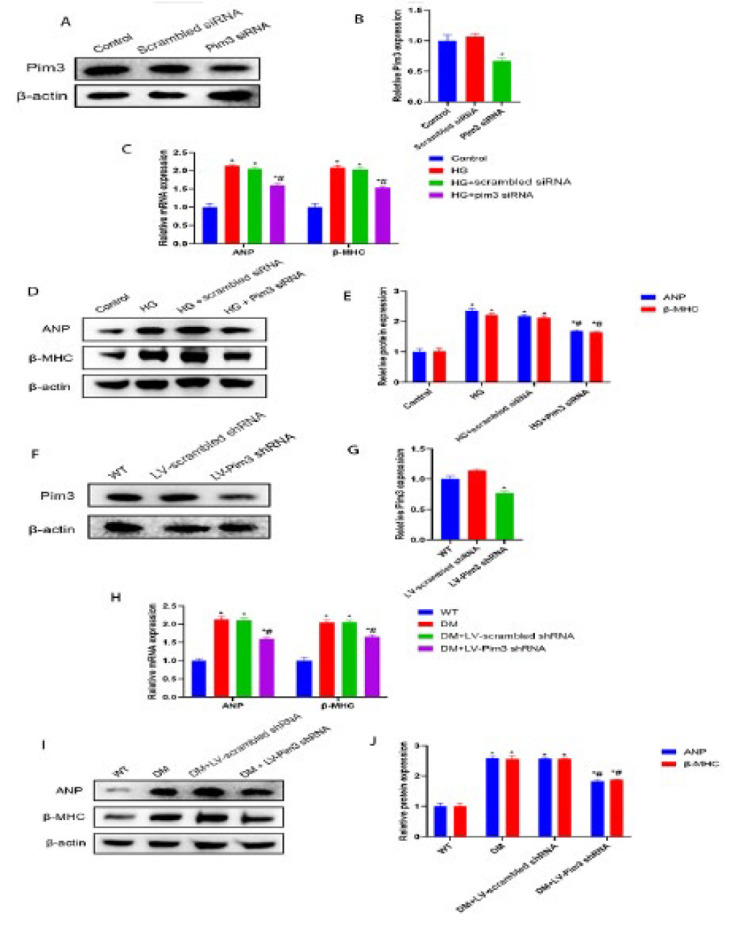
Attenuating the mRNA and protein expression of hyperglycemia-induced cardiac hypertrophy markers by Pim3 silencing

(A) After treatment with or without scrambled siRNA or Pim3 siRNA in H9C2 cells, changes in Pim3 protein expression were detected by western blotting. (C) H9C2 cells were either transfected with scrambled siRNA or Pim3 siRNA and exposed to HG for 48 hr. The mRNA expression of ANP and β-MHC was measured by RT-qPCR, and quantitative analysis is shown in the histogram. (D) In cells treated as described in (C), the protein expression of ANP and β-MHC in H9C2 cells was evaluated by western blotting. (F) Treatment with or without LV-scrambled or LV-Pim3 shRNA in mice changed Pim3 protein expression, as detected by western blotting. (H) After treatment of WT or diabetic mice with or without LV-scrambled/LV-Pim3 shRNA, the mRNA expression of ANP and β-MHC was measured by RT-PCR, and quantitative analysis is shown in the histogram. (I) After the treatments described in (H), the protein expression of ANP and β-MHC in heart tissues was measured by western blotting. (B, E, G, J) Quantitative presentation of immunoblots from western blotting is displayed. All data were obtained from at least three independent experiments. *P<0.05 vs control or WT. #*P<*0.05 vs HG/HG+scrambled siRNA or DM/DM+LV-scrambled shRNA

WT, wild-type mice; LV-scrambled shRNA, WT mice treatment with LV-scrambled shRNA; LV-Pim3 shRNA, WT mice treatment with LV-Pim3 shRNA; DM, diabetic mice; DM+LV-scrambled shRNA,diabetic mice treatment with LV-scrambled shRNA;

DM+LV-Pim3 shRNA, diabetic mice treatment with LV-Pim3 shRNA

**Table 2:** Serum NT-proBNP concentration in diabetic mice treated with or without LV-Pim3 shRNA for 12 weeks

	WT	DM	DM+LV-scrambled shRNA	DM+LV-Pim3 shRNA
NT-proBNP (pg/ml)	115.06 ± 59.11	298.03 ± 62.11^**^	284.23 ± 51.43^**^	202.50 ± 53.45^*#^

n=8 in each group, *P<0.05, **P<0.01 vs WT; ^#^ *P<*0.05 vs DM or DM+LV-scrambled shRNA

Wild-type mice, WT; DM, diabetes mice; Lentivirus, LV; NT-proBNP, N-terminal pro-brain natriuretic peptide

**Figure 3:**
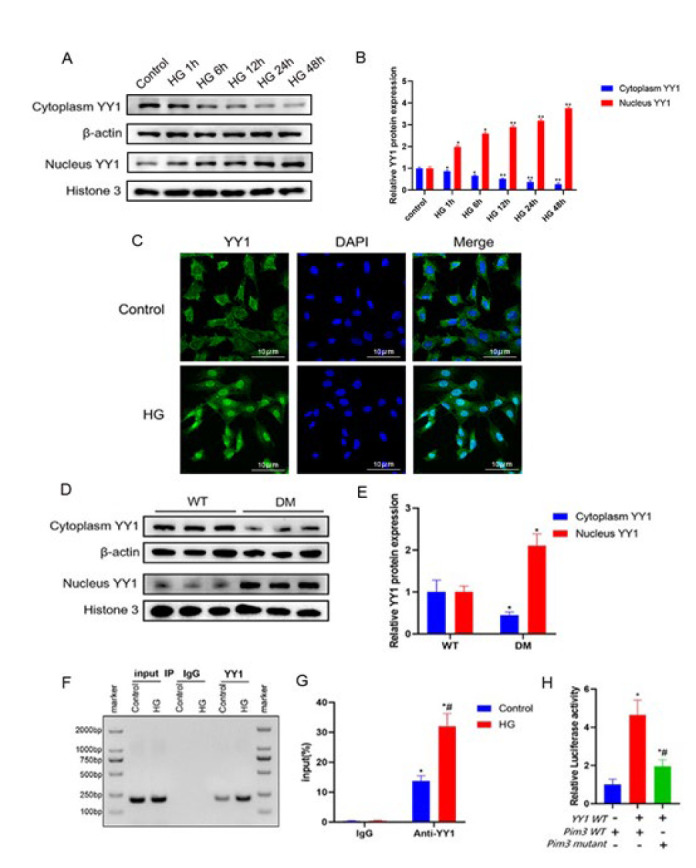
Improvements in diabetes-induced cardiac hypertrophy and cardiac dysfunction after Pim3 silencing

(A, B) After treating diabetic mice with or without LV-Pim3 shRNA for 12 weeks, cardiomyocyte size was evaluated by WGA staining (A), and quantitative analysis is shown in the histogram (B). (C, D) After applying the treatments described in (A), representative M-mode echocardiograms were obtained for each group (C). The column figures show the differences in left ventricular EF and FS (D). n=6 in each group. *P<0.05 vs WT; ^#^ *P<*0.05 vs DM or DM+LV-scrambled shRNA

WT, wild-type mice; DM, diabetic mice; DM+LV-scrambled shRNA,diabetic mice treatment with LV-scrambled shRNA; DM+LV-Pim3 shRNA, diabetic mice treatment with LV-Pim3 shRNA

**Figure 4:**
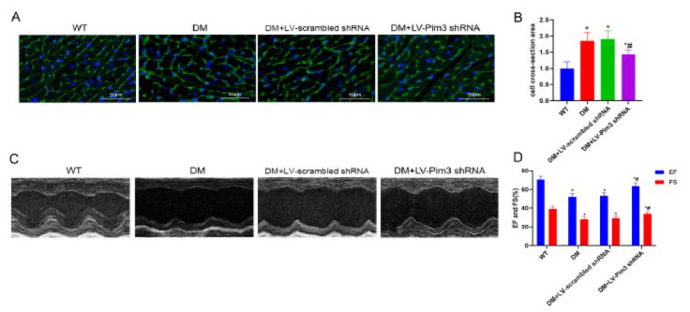
Regulation of Pim3 by YY1 under hyperglycemic stimulation

(A, B) H9C2 cells were treated with or without HG for the indicated times, and YY1 translocation from the cytosol to the nucleus in vitro was measured by western blotting (A) and is displayed quantitatively as immunoblots (B). *P<0.01, **P<0.01 vs control. (C) H9C2 cells were exposed to HG for 48 hr, and YY1 translocation from the cytosol to the nucleus in vitro was determined by immunofluorescence. (D, E) YY1 translocation from the cytosol to the nucleus in heart tissues from WT or diabetic mice was determined by western blotting (D) and is displayed quantitatively as immunoblots (E). n = 6 in each group. *P<0.05 vs WT. (F, G) HL-1 cells were treated with or without HG for 48 hr, and crosslinked chromatin was extracted and immunoprecipitated with anti-YY1 or IgG antibody. Immunoprecipitated DNA was amplified by PCR using mouse Pim3 promoter-specific primers. PCR products from a representative experiment are shown on the left (F), whereas the band intensities are shown on the right (G). *P<0.05 vs IgG group, ^# ^*P<*0.05 vs control. (H) HL-1 cells were co-transfected with the YY1 expression vector and Pim3 WT or Pim3 mutant. Twenty-four hours after transfection, cell lysates were prepared for the luciferase assay. The data represent relative Pim3 promoter activity normalized to Renilla luciferase activity. *P<0.05 vs Pim3 WT, ^#^*P<*0.05 vs YY1 WT/Pim3 WT. All data were obtained from three independent experiments

HG, high glucose; Wild-type mice, WT; DM, diabetes mice; YY1 WT, YY1 expression vector; Pim3 WT, Pim3 promoter-reporter plasmid; Pim3 mutant, Pim3 promoter-reporter mutant, namely mutation of the YY1 binding site in the Pim3 promoter

**Figure 5:**
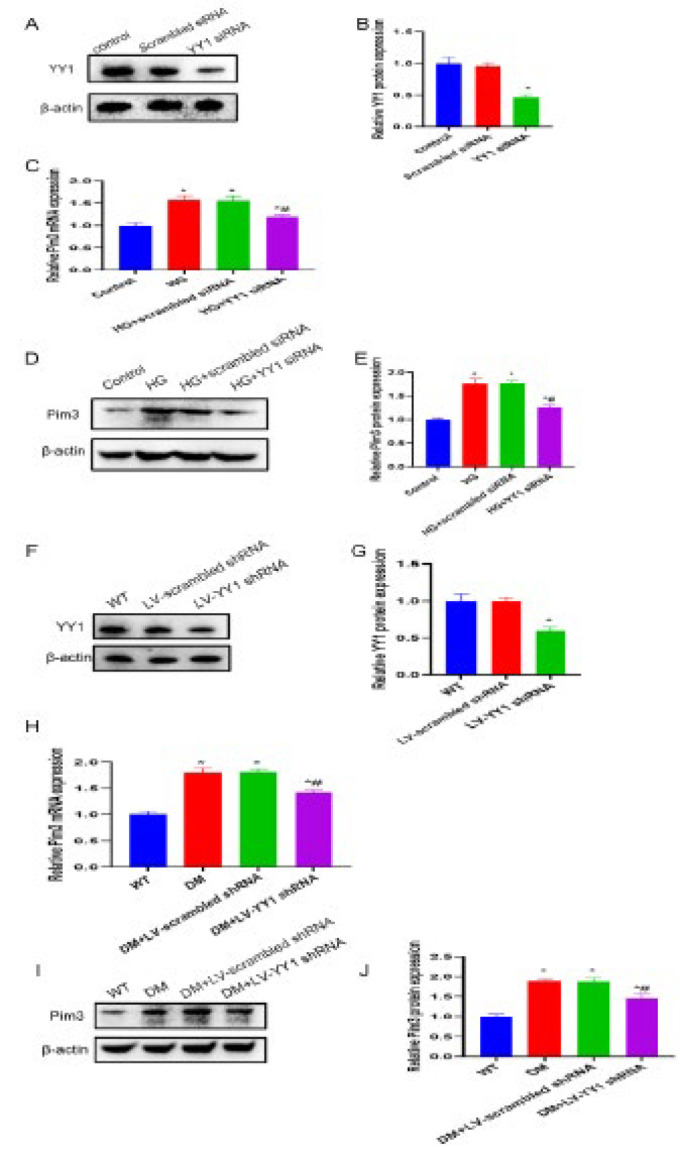
Attenuation of hyperglycemia-induced Pim3 mRNA and protein expression by YY1 silencing

(A) After treatment with or without scrambled or YY1 siRNA in H9C2 cells, changes in YY1 protein expression were measured by western blotting. (C) H9C2 cells were either transfected with scrambled or YY1 siRNA and exposed to HG for 48 hr. The mRNA expression of Pim3 was measured by RT-qPCR. (D) In cells treated as described in (C), Pim3 protein expression in H9C2 cells was measured by western blotting. (F) After treatment with or without LV-scrambled shRNA or LV-YY1 shRNA in mice, changes in YY1 protein expression were measured by western blotting. (H) After WT or diabetic mice were treated with or without LV-scrambled shRNA/LV-YY1 shRNA for twelve weeks, the mRNA expression of Pim3 was measured by RT-PCR, and quantitative analysis is shown in the histogram. (I) After the treatment described in (H), Pim3 protein expression in the heart tissue was measured by western blotting. (B, E, G, J) Quantitative immunoblots from western blotting are displayed. All data were obtained from at least three independent experiments. *P<0.05 vs control or WT. ^# ^*P<*0.05 vs HG/HG+scrambled siRNA or DM/DM+LV-scrambled shRNA

WT, wild-type mice; LV-scrambled shRNA, WT mice treatment with LV-scrambled shRNA; LV-YY1 shRNA, WT mice treatment with LV-YY1 shRNA; DM, diabetic mice; DM+LV-scrambled shRNA,diabetic mice treatment with LV-scrambled shRNA;

DM+LV-YY1 shRNA, diabetic mice treatment with LV-YY1 shRNA

**Figure 6:**
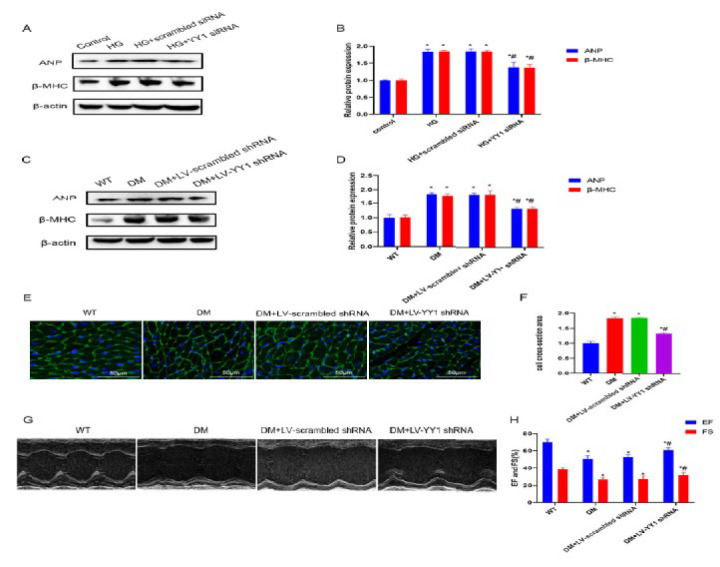
Improvement in hyperglycemia-induced cardiac hypertrophy and dysfunction by YY1 silencing

(A) H9C2 cells were transfected with either scrambled or YY1 siRNA and exposed to HG medium for 48 hr. ANP and β-MHC protein expression were measured by western blotting. (C) After WT or diabetic mice were treated with or without LV-YY1 shRNA for twelve weeks, the protein expression of ANP and β-MHC was evaluated by Western blotting. (B, D) Quantitative immunoblots from western blotting are displayed. All data were obtained from at least three independent experiments. *P<0.05 vs control or WT. #*P<*0.05 vs HG/HG+scrambled siRNA or DM/DM+LV scrambled shRNA (E, F) After applying the treatments described in (C), cardiomyocyte size was evaluated by WGA staining (E), and the quantitative analysis is shown as a histogram (F). (G, H) After applying the treatments described in (C), M-mode echocardiograms were obtained, and representative examples are shown for each group (G). The differences in left. Ventricular EF and FS are shown in the column figures (H). n=6 in each group. *P<0.05 vs WT; ^#^*P<*0.05 vs DM or DM+LVscrambled

shRNA

WT, wild-type mice; DM, diabetic mice; DM+LV-scrambled shRNA,diabetic mice treatment with LV-scrambled shRNA; DM+LV-YY1 shRNA, diabetic mice treatment with LV-YY1 shRNA

**Table 3:** Serum NT-proBNP in diabetic mice treated with or without LV-YY1 shRNA for twelve weeks

	WT	DM	DM+LV-scrambled shRNA	DM+LV-YY1 shRNA
NT-proBNP (pg/ml)	110.71 ± 47.22	302.67 ± 55.80^**^	296.03 ± 60.18^**^	200.48 ± 45.69^*#^

Wild-type mice, WT; DM, diabetes mice; Lentivirus, LV; NT-proBNP, N-terminal pro-brain natriuretic peptide. n = 6 in each group, *P<0.05, **P<0.01 vs WT; ^#^*P<*0.05 vs DM or DM+LV-scrambled shRNA

## Results

***Hyperglycemia up-regulates Pim3 protein expression in H9C2 cells and diabetic hearts***

Compared with the control group or wild-type (WT) mice, Pim3 RNA expression was significantly up-regulated in both HG-cultured H9C2 cells and diabetic hearts (both *P*<0.05)(Figure 1A, 1B). To determine the effect of hyperglycemia on Pim3 protein expression, H9C2 cells were exposed to HG (33 mM) for 0-48 hr, and Pim3 protein expression was analyzed by western blotting. The results showed that Pim3 protein expression was significantly up-regulated after 1 hr of HG treatment (*P*<0.05) and maintained for up to 48 hr, with a peak at 48 hr (*P*<0.01) compared with the control group (Figure 1C, 1D). These effects were not observed in H9C2 cells exposed to mannitol (Figure 1E, 1F). The data indicate that hyperosmosis did not exert an effect on Pim3 protein expression. Moreover, compared with the WT group, Pim3 protein expression was significantly up-regulated in diabetic hearts at 2 months (*P*<0.05) and peaked at 3 months (*P*<0.01) in mice with streptozotocin-induced DM (Figure 1G, 1H). As mentioned above, Pim3 protein expression was up-regulated under hyperglycemic stimulation. 

***Pim3 knockdown attenuates hyperglycemia-induced cardiac hypertrophy and HF***

To determine whether Pim3 is involved in hyperglycemia-induced cardiac hypertrophy, the mRNA and protein expression of cardiac hypertrophy markers was analyzed, both in HG-treated H9C2 cells and in diabetic hearts after knockdown of Pim3. Compared with the control or scrambled siRNA groups, Pim3 protein expression was reduced by approximately 68% after H9C2 cells were transfected with Pim3 siRNA (Pim3 siRNA vs control: 0.3133±0.0416 vs 1.013±0.1007; *P*<0.05)(Figure 2A, 2B). Furthermore, compared with the control group, the mRNA and protein expression of cardiac hypertrophy markers, such as ANP and β-MHC, was significantly up-regulated when H9C2 cells were exposed to HG (*P*<0.05). However, treatment with Pim3 siRNA, but not scrambled siRNA, significantly attenuated hyperglycemia-induced effects (*P*<0.05)(Figure 2C, 2D, 2E). Next, we evaluated the knockdown effect of lentivirus (LV)-mediated Pim3 shRNA. Compared with WT mice, Pim3 protein expression was reduced by around 54% after the mice were treated with LV-Pim3 shRNA (LV-Pim3 shRNA vs WT: 0.4733±0.055 vs 1.027±0.1124; *P*<0.05)(Figure 2F, 2G). The mRNA and protein expression of both ANP and β-MHC was higher in diabetic mice than in WT mice (both *P*<0.05), while these DM-induced effects were significantly mitigated at twelve weeks after tail vein injection of LV-Pim3 shRNA (*P*<0.05) (Figure 2H, 2I, 2J). Moreover, diabetic mice showed a larger cardiomyocyte size and higher serum NT-proBNP than WT mice (both *P*<0.05). Administration of LV-Pim3 shRNA for 12 weeks, but not LV-scrambled shRNA, markedly reduced cell size (Figure 3A, 3B) and decreased NT-proBNP (Table 2) in diabetic mice (*P*<0.05). Echocardiography showed lower ejection fraction (EF) (DM vs WT: 51.00±2.64 vs 67.77±2.35; *P*<0.05) and fractional shortening (FS)(DM vs WT: 28.20±1.80 vs 39.07±2.20; *P*<0.05) in diabetic mice than in WT mice (both *P*<0.05), and treatment with LV-Pim3 shRNA, but not LV-scrambled shRNA, significantly increased EF (DM+LV-Pim3 shRNA vs DM: 63.17±1.25 vs 51.67±2.51; *P*<0.05) and FS (DM+LV-Pim3 shRNA vs DM: 33.60±1.05 vs 28.67±1.72; *P*<0.05) (both *P*<0.05)(Figure 3C, 3D). Taken together, these data indicate that Pim3 is involved in hyperglycemia-induced cardiac hypertrophy and HF.

***YY1 nuclear translocation in cardiomyocytes causes hyperglycemia-induced Pim3 up-regulation***

As mentioned above, Pim3 RNA expression was markedly up-regulated in both HG-cultured H9C2 cells and diabetic hearts. This showed that hyperglycemia increased Pim3 expression at the RNA level. Thus, we proposed that hyperglycemia might be involved in the transcriptional regulation of Pim3 by targeting transcription factors. To test this hypothesis, we assessed the transcription factors that bind to the Pim3 promoter in the JASPAR database (http://jaspar.genereg.net/). YY1, which is a transcriptional repressor or activator, was predicted to be one of the most promising candidates. As shown in Figure 4A and 4B, the amount of YY1 that translocated from the cytosol to the nucleus increased after 1 hr of HG treatment (*P*<0.05) and peaked at 48 hr (*P*<0.01) compared with the control group. Immunofluorescence also confirmed that HG induced YY1 nuclear translocation (Figure 4C). In addition, compared with WT mice, YY1 nuclear translocation was significantly enhanced in diabetic hearts (*P*<0.05)(Figure 4D, 4E). Further analysis via ChIP-PCR demonstrated that YY1 binding to the Pim3 promoter was intensified when HL-1 cells were stimulated with HG compared with the control group (*P*<0.05)(Figure 4F, 4G). Luciferase activity was enhanced in HL-1 cells co-transfected with the YY1 vector and the Pim3 promoter-reporter plasmid compared with cells treated with YY1 and the Pim3 promoter-reporter mutant plasmid (*P*<0.05)(Figure 4H). These findings suggest that hyperglycemia induced YY1 nuclear translocation in cardiomyocytes and enhanced Pim3 transcriptional activity, eventually contributing to Pim3 mRNA up-regulation.

***YY1 knockdown reduces hyperglycemia-induced Pim3 expression in vitro and in vivo***

Compared with the control or scrambled siRNA group, YY1 protein expression was reduced by approximately 70% after H9C2 cells were transfected with YY1 siRNA (YY1 siRNA vs control: 0.3033±0.0351 vs 1.033±0.1235; *P*<0.05)(Figure 5A, 5B). Furthermore, the mRNA and protein expression of Pim3 was significantly up-regulated when H9C2 cells were exposed to HG compared with the control group (*P*<0.05), while treatment with YY1 siRNA, but not scrambled siRNA, markedly attenuated these hyperglycemia-induced effects (*P*<0.05)(Figure 5C, 5D, 5E). In addition, the knockdown effect of LV-mediated YY1 shRNA was evaluated. Compared with WT mice, YY1 protein expression was reduced by around 55% after mice were treated with LV-YY1 shRNA (LV-YY1 shRNA vs WT: 0.46±0.0755 vs 0.9767±0.0873; *P*<0.05)(Figure 5F, 5G). Pim3 mRNA and protein expression were significantly higher in diabetic mice than in WT mice (*P*<0.05). However, this diabetes-induced effect was significantly attenuated at 12 weeks after treatment with LV-YY1 shRNA (*P*<0.05)(Figure 5H, 5I, 5J). Collectively, YY1 mediated hyperglycemia-induced Pim3 expression, both in HG-treated H9C2 cells and in streptozotocin-induced diabetic hearts.

***YY1 knockdown attenuates hyperglycemia-induced cardiac hypertrophy and HF***

As shown in Figures 6A and 6B, the protein expression of ANP and β-MHC was significantly higher in HG-treated H9C2 cells than in the control group (*P*<0.05), while treatment with YY1 siRNA, but not scrambled siRNA, mitigated this hyperglycemia-induced effect (*P*<0.05). Similarly, ANP and β-MHC protein expression was significantly higher in diabetic mice than in WT mice (*P*<0.05), while this diabetes-induced effect was attenuated after treatment with LV-YY1 shRNA for twelve weeks (*P*<0.05)(Figure 6C, 6D). Furthermore, compared with WT mice, increased cardiomyocyte size and higher serum NT-proBNP were observed in diabetic mice. However, treatment with LV-YY1 shRNA for 12 weeks, but not LV-scrambled shRNA, markedly reduced the cell size (Figure 6E, 6F) and decreased NT-proBNP (Table 3) in diabetic mice (*P*<0.05). Echocardiography showed lower EF (DM vs WT: 50.93±1.40 vs 69.33±2.08; *P*<0.05) and FS (DM vs WT: 27.30±1.08 vs 38.23±1.16; *P*<0.05) in diabetic mice than in WT mice (*P*<0.05), and administration of LV-YY1 shRNA, but not LV-scrambled shRNA, significantly increased EF (DM+LV-YY1 shRNA vs DM: 62.17±1.75 vs 51.83±2.02; *P*<0.05) and FS (DM+LV-YY1 shRNA vs DM: 32.73±1.32 vs 28.17±0.96; *P*<0.05)(*P*<0.05)(Figure 6G, 6H). Taken together, these data indicate that YY1 is involved in hyperglycemia-induced cardiac hypertrophy and HF.

## Discussion

HF is a global health issue that lacks effective curative therapies, with existing therapies only providing symptom relief or management. Many risk factors, including DM, are involved in the pathogenesis of chronic HF (26). DM is associated with a 2–4-fold increase in the risk of HF (4). Unfortunately, even tight glycemic control has not improved DM-induced HF. Although the specific mechanism through which DM causes HF is not well understood, DM-induced cardiac hypertrophy as a cause of HF has been reported (5). Therefore, inhibiting hyperglycemia-induced cardiac hypertrophy could improve cardiac function under DM conditions. In this study, we demonstrated that hyperglycemia-induced sustained YY1 nuclear translocation strengthened the binding of YY1 to the Pim3 promoter and markedly enhanced Pim3 promoter transcription activity, leading to Pim3 up-regulation, eventually contributing to cardiac hypertrophy and HF.

 Cardiac hypertrophy is characterized by cardiomyocyte enlargement, increased protein synthesis, fetal gene reactivation, cytoskeleton remodeling, and enhanced fibrosis (2). Although molecular mechanisms have been studied at multiple levels, feasible targets for preventing or reversing the progression of cardiac hypertrophy are still scarce. Proto-oncogenes, such as c-Myc and c-Fos, are known to modulate myocardial hypertrophy (9). Pim kinases, including Pim1, Pim2, and Pim3, are proto-oncogenes with serine/threonine kinase activity (12). Accumulating evidence suggests that Pim1 exerts protective effects on diabetes-induced myocardial injury and is down-regulated in diabetic hearts (13-15). Another study demonstrated that Pim1 contributes to myocardial hypertrophy in mice after transverse aortic constriction (27). Wang *et al*. reported that the Pim3 protein was increased in a time-dependent manner when vascular smooth muscle was exposed to HG (28). These findings indicate that the expression of Pim1 varies among different animal models and cell types. Theoretically, Pim3, which has a similar structure to Pim1, should play a central role in the pathogenesis of DM-induced myocardial hypertrophy and HF. This study found that Pim3 RNA and protein expression were significantly up-regulated in diabetic hearts and HG-cultured H9C2 cells. Pim3 knockdown markedly attenuated myocardial hypertrophy and cardiac dysfunction. Our data suggest that targeting Pim3 may be effective for treating DM-induced myocardial hypertrophy and HF. Given that reciprocal compensation has been reported in mouse models with knockout of all three Pim kinases (Pim triple-knockout)(29, 30), it is speculated that the discrepancy in expression between Pim1 and Pim3 under DM conditions may be attributed to Pim3 compensation for Pim1 loss. The transcriptome of diabetic hearts in Gene Expression Omnibus datasets (GSE6880, GSE36875) and previous literature (31) demonstrates up-regulated Pim3. Thus, exploring HG-induced Pim3 up-regulation is of great significance. 

In the JASPAR database (http://jaspar.genereg.net/), YY1 was predicted to be one of the most promising transcription factors that bind to the Pim3 promoter. YY1 is a ubiquitously expressed and evolutionarily conserved zinc finger protein with a pivotal role in normal biological processes, such as cell development, differentiation, replication, and proliferation, and it is associated with several pathological conditions (18, 19). YY1 protein abundance significantly increases in failing human hearts and transgenic mice with hypertrophic cardiomyopathy (20, 32). Therefore, in this study, we postulated that YY1 would mediate DM-associated cardiac hypertrophy and subsequent HF. Indeed, as we expected, YY1 was involved in hyperglycemia-induced Pim3 up-regulation in cardiomyocytes by binding to the Pim3 promoter and markedly enhancing Pim3 promoter transcription activity, eventually contributing to cardiac hypertrophy and HF. Knockdown of YY1 mitigated these effects. 

However, the role of YY1 in heart disease is controversial. YY1 ameliorates cardiac injury and remodeling after myocardial infarction (33). YY1 also protects cardiomyocytes from pathological hypertrophy in response to hypertrophic stimuli (34). The same research group showed that overexpression of YY1 in a transgenic mouse model induced pathologic cardiac hypertrophy (20). Whether YY1 functions as an antihypertrophic or hypertrophic factor may depend on the specific disease process. Acute-phase YY1 exerts protection, whereas chronic duration results in pathology. Diabetes-induced cardiac hypertrophy and HF are chronic processes (4); therefore, it is reasonable that YY1 functions as a hypertrophic factor under these conditions. Non-cardiomyocytes also play a pivotal role in DM-induced cardiac hypertrophy (35). However, whether targeting the YY1-Pim3 axis in non-cardiomyocytes can exert antihypertrophic and cardioprotective effects remains unknown, which is one of the study’s limitations.

## Conclusion

Under hyperglycemic conditions, sustained YY1 nuclear translocation induced significant Pim3 up-regulation, eventually leading to cardiac hypertrophy and HF in mice. Targeting the YY1-Pim3 axis markedly improved these effects. The present study provides exciting insights into establishing novel therapies for DM-induced HF.

## Data Availability

The raw data supporting the conclusions of this article are available from the corresponding author upon reasonable request.

## Declaration

We have not used any AI tools or technologies to prepare this manuscript.

## References

[1] Ge W, Hou C, Zhang W, Guo X, Gao P, Song X (2021). Mep1a contributes to Ang II-induced cardiac remodeling by promoting cardiac hypertrophy, fibrosis and inflammation. J Mol Cell Cardiol.

[2] Zhang Y, Da Q, Cao S, Yan K, Shi Z, Miao Q (2021). HINT1 (histidine triad nucleotide-binding protein 1) attenuates cardiac hypertrophy via suppressing HOXA5 (Homeobox A5) expression. Circulation.

[3] Tang X, Pan L, Zhao S, Dai F, Chao M, Jiang H (2020). SNO-MLP (S-nitrosylation of muscle LIM protein) facilitates myocardial hypertrophy through TLR3 (toll-like receptor 3)-mediated RIP3 (receptor-interacting protein kinase 3) and NLRP3 (NOD-like receptor pyrin domain containing 3) inflammasome activation. Circulation.

[4] Park JJ (2021). Epidemiology, pathophysiology, diagnosis and treatment of heart failure in diabetes. Diabetes Metab J.

[5] Cibi DM, Sandireddy R, Bogireddi H, Tee N, Ghani S, Singh BK (2021). Cardiac tissue factor regulates inflammation, hypertrophy, and heart failure in mouse model of type 1 diabetes. Diabetes.

[6] Xu Q, Ding H, Li S, Dong S, Li L, Shi B (2021). Sleeve gastrectomy ameliorates diabetes-induced cardiac hypertrophy correlates with the MAPK signaling pathway. Front Physiol.

[7] Asghari AA, Mahmoudabady M, Mousavi Emadi Z, Hosseini SJ, Salmani H (2022). Cardiac hypertrophy and fibrosis were attenuated by olive leaf extract treatment in a rat model of diabetes. J Food Biochem.

[8] Farazandeh M, Mahmoudabady M, Asghari AA, Niazmand S (2022). Diabetic cardiomyopathy was attenuated by cinnamon treatment through the inhibition of fibro-inflammatory response and ventricular hypertrophy in diabetic rats. J Food Biochem.

[9] Oceandy D, Cartwright EJ, Neyses L (2009). Ras-association domain family member 1A (RASSF1A)-where the heart and cancer meet. Trends Cardiovasc Med.

[10] Liu DX, Liu XM, Su Y, Zhang XJ (2011). Renal expression of proto-oncogene Ets-1 on matrix remodeling in experimental diabetic nephropathy. Acta Histochem.

[11] Kowluru RA, Kowluru A, Chakrabarti S, Khan Z (2004). Potential contributory role of H-Ras, a small G-protein, in the development of retinopathy in diabetic rats. Diabetes.

[12] Mikkers H, Nawijn M, Allen J, Brouwers C, Verhoeven E, Jonkers J (2004). Mice deficient for all PIM kinases display reduced body size and impaired responses to hematopoietic growth factors. Mol Cell Biol.

[13] Katare RG, Caporali A, Oikawa A, Meloni M, Emanueli C, Madeddu P (2010). Vitamin B1 analog benfotiamine prevents diabetes-induced diastolic dysfunction and heart failure through Akt/Pim-1-mediated survival pathway. Circ Heart Fail.

[14] Katare R, Caporali A, Zentilin L, Avolio E, Sala-Newby G, Oikawa A (2011). Intravenous gene therapy with PIM-1 via a cardiotropic viral vector halts the progression of diabetic cardiomyopathy through promotion of prosurvival signaling. Circ Res.

[15] Moore A, Shindikar A, Fomison-Nurse I, Riu F, Munasinghe PE, Ram TP (2014). Rapid onset of cardiomyopathy in STZ-induced female diabetic mice involves the downregulation of pro-survival Pim-1. Cardiovasc Diabetol.

[16] Xu Y, Xing Y, Xu Y, Huang C, Bao H, Hong K (2016). Pim-2 protects H9c2 cardiomyocytes from hypoxia/reoxygenation-induced apoptosis via downregulation of Bim expression. Environ Toxicol Pharmacol.

[17] Yao M, Li L, Huang M, Tan Y, Shang Y, Meng X (2021). Sanye tablet ameliorates insulin resistance and dysregulated lipid metabolism in high-fat diet-induced obese mice. Front Pharmacol.

[18] Gordon S, Akopyan G, Garban H, Bonavida B (2006). Transcription factor YY1: Structure, function, and therapeutic implications in cancer biology. Oncogene.

[19] Asensio-Lopez MC, Lax A, Fernandez del Palacio MJ, Sassi Y, Hajjar RJ, Januzzi JL (2019). Yin-Yang 1 transcription factor modulates ST2 expression during adverse cardiac remodeling post-myocardial infarction. J Mol Cell Cardiol.

[20] Stauffer BL, Dockstader K, Russell G, Hijmans J, Walker L, Cecil M (2015). Transgenic over-expression of YY1 induces pathologic cardiac hypertrophy in a sex-specific manner. Biochem Biophys Res Commun.

[21] Fujino M, Morito N, Hayashi T, Ojima M, Ishibashi S, Kuno A (2023). Transcription factor c-Maf deletion improves streptozotocin-induced diabetic nephropathy by directly regulating Sglt2 and Glut2. JCI Insight.

[22] Zuo GF, Wang LG, Huang L, Ren YF, Ge Z, Hu ZY (2024). TAX1BP1 downregulation by STAT3 in cardiac fibroblasts contributes to diabetes-induced heart failure with preserved ejection fraction. Biochim Biophys Acta Mol Basis Dis.

[23] Nan J, Hu H, Sun Y, Zhu L, Wang Y, Zhong Z (2017). TNFR2 stimulation promotes mitochondrial fusion via Stat3- and NF-kB-dependent activation of OPA1 expression. Circ Res.

[24] Zhou S, Dai Q, Huang X, Jin A, Yang Y, Gong X (2021). STAT3 is critical for skeletal development and bone homeostasis by regulating osteogenesis. Nat Commun.

[25] Liu Z, Yao X, Yan G, Xu Y, Yan J, Zou W (2016). Mediator MED23 cooperates with RUNX2 to drive osteoblast differentiation and bone development. Nat Commun.

[26] Triposkiadis F, Xanthopoulos A, Parissis J, Butler J, Farmakis D (2022). Pathogenesis of chronic heart failure: Cardiovascular aging, risk factors, comorbidities, and disease modifiers. Heart Fail Rev.

[27] Lei B, Chess DJ, Keung W, O’Shea KM, Lopaschuk GD, Stanley WC (2008). Transient activation of p38 MAP kinase and up-regulation of Pim-1 kinase in cardiac hypertrophy despite no activation of AMPK. J Mol Cell Cardiol.

[28] Wang K, Deng X, Shen Z, Jia Y, Ding R, Li R (2017). High glucose promotes vascular smooth muscle cell proliferation by upregulating proto-oncogene serine/threonine-protein kinase Pim-1 expression. Oncotarget.

[29] Din S, Konstandin MH, Johnson B, Emathinger J, Volkers M, Toko H (2014). Metabolic dysfunction consistent with premature aging results from deletion of Pim kinases. Circ Res.

[30] Beharry Z, Mahajan S, Zemskova M, Lin YW, Tholanikunnel BG, Xia Z (2011). The Pim protein kinases regulate energy metabolism and cell growth. Proc Natl Acad Sci U S A.

[31] van Lunteren E, Moyer M (2007). Oxidoreductase, morphogenesis, extracellular matrix, and calcium ion-binding gene expression in streptozotocin-induced diabetic rat heart. Am J Physiol Endocrinol Metab.

[32] Sucharov CC, Mariner P, Long C, Bristow M, Leinwand L (2003). Yin Yang 1 is increased in human heart failure and represses the activity of the human alpha-myosin heavy chain promoter. J Biol Chem.

[33] Huang Y, Li L, Chen H, Liao Q, Yang X, Yang D (2021). The protective role of yin-yang 1 in cardiac injury and remodeling after myocardial infarction. J Am Heart Assoc.

[34] Sucharov CC, Dockstader K, McKinsey TA (2008). YY1 protects cardiac myocytes from pathologic hypertrophy by interacting with HDAC5. Mol Biol Cell.

[35] Phang RJ, Ritchie RH, Hausenloy DJ, Lees JG, Lim SY (2023). Cellular interplay between cardiomyocytes and non-myocytes in diabetic cardiomyopathy. Cardiovasc Res.

